# Expression of Concern: Identification of the Sites of Tau Hyperphosphorylation and Activation of Tau Kinases in Synucleinopathies and Alzheimer’s Diseases

**DOI:** 10.1371/journal.pone.0295638

**Published:** 2023-12-05

**Authors:** The PLOS ONE Editors

After this article [1] was published, concerns were raised regarding Figs 1 and 2.

Specifically:

- In Fig 1A, the Thr217 control bands appear similar to the Ser238 control bands.
- In Fig 1C, the Ser235 AD bands appear similar to the Ser413 AD bands.
- In Fig 2A, there appear to be vertical discontinuities between all bands in the α-Syn panel and between the Control and PD-FC bands in the γ-Syn panel.
- In Fig 2B, the second “control” band in the β-Syn panel appears to be flanked by vertical discontinuities.

The authors did not respond to the concerns that were raised regarding these figures, and no underlying data was provided for editorial review. Accordingly these concerns cannot be resolved and the *PLOS ONE* Editors remain concerned about the issues contained within these figures.

In light of the above concerns, the *PLOS ONE* Editors issue this Expression of Concern.

All authors either did not respond directly or could not be reached.
